# Antiplatelet therapy and coronary artery bypass grafting: Protocol for a systematic review and network meta-analysis

**DOI:** 10.1097/MD.0000000000016880

**Published:** 2019-08-23

**Authors:** Saurabh Gupta, Emilie P. Belley-Côté, Bram Rochwerg, Anthony Bozzo, Puru Panchal, Arjun Pandey, Lawrence Mbuagbaw, Shamir Mehta, J-D. Schwalm, Richard P. Whitlock

**Affiliations:** a Department of Surgery; b Department of Health Research Methods, Evidence and Impact; c Department of Medicine; d Faculty of Health Sciences, McMaster University; e Population Health Research Institute, Hamilton, Ontario, Canada.

**Keywords:** antiplatelet therapy, cardiac surgery, coronary artery bypass grafting, coronary artery disease, network meta-analysis

## Abstract

**Introduction:**

Saphenous vein graft (SVG) is the most common conduit used for coronary artery bypass grafting (CABG) surgery. Unfortunately, SVG are associated with poor long-term patency rates; a significant predictor of re-operation rates and survival. As such, medical therapy to prevent SVG narrowing or occlusion is of paramount importance. Aspirin (ASA) monotherapy is the standard of care after CABG, to improve long-term major adverse cardiovascular events (MACE) and graft patency. Benefits of dual antiplatelet therapy (DAPT) have not been well established in all CABG patients. We present a protocol for a network meta-analysis (NMA) comparing the effects of various antiplatelet therapy regimens on SVG patency, mortality, and bleeding among adult patients following CABG.

**Methods:**

We will search CENTRAL, MEDLINE, EMBASE, CINAHL ACPJC, and grey literature sources (AHA, ACC, ESC, and CCC conference proceedings, ISRCTN Register, and WHO ICTRP) for randomized controlled trials (RCTs) which fit our criteria. RCTs that evaluate different antiplatelet regimens at least 3-months after CABG and have any of SVG patency, mortality, MACE, and major bleeding as outcomes will be selected. We will perform title and abstract screening, full-text screening, and data extraction independently and in duplicate. Two independent reviewers will also assess risk of bias (ROB) for each study, as well as evaluate quality of evidence using the GRADE framework. We will use R to perform the NMA and use low-dose ASA as reference within our network. We will report results as odds ratios with confidence intervals for direct comparisons, and credible intervals for indirect or mixed comparisons. We will use the surface under the cumulative ranking curve (SUCRA) to estimate the ranking of interventions.

**Discussion:**

Given the limited direct comparison of various antiplatelet regimens, a network approach is ideal to clarify the optimum antiplatelet therapy after CABG. We hope that our NMA will be the largest quantitative synthesis evaluating antiplatelet regimens among patients requiring CABG. It should inform clinicians and guideline developers in selecting the most effective and safest antiplatelet regimen.

**Systematic Review registration:** International Prospective Register for Systematic Reviews (PROSPERO)—CRD42019127695.

## Abstract 2

Supplemental Digital Content is available in the text

## 1 Introduction

Saphenous vein graft (SVG) remains the most commonly used conduit for coronary artery bypass grafting (CABG) surgery, with patency rates as low as 50% at 10 years.^[1,2]^ Graft patency is a significant predictor of re-operation rates and survival; therefore, medical therapy to prevent SVG narrowing or occlusion is of paramount importance.^[3,4]^

Antiplatelet therapy, specifically acetylsalicylic acid (ASA), is standard of care after CABG, and has repeatedly been demonstrated to improve long-term major adverse cardiovascular events (MACE) and graft patency.^[5–8]^ Based on data from large randomized controlled trials (RCTs), dual antiplatelet therapy (DAPT)—a combination of ASA and P2Y12 inhibitors—is recommended for patients with acute coronary syndrome (ACS) regardless of revascularization modality.^[9–11]^ More potent antiplatelet agents (P2Y12 inhibitors; clopidogrel, prasugrel, and ticagrelor) have been evaluated in patients undergoing CABG after an ACS. A meta-analysis of RCTs and sub-groups of larger RCTs analyzed 4887 CABG patients, suggested a significant reduction in all-cause mortality using DAPT with ASA and ticagrelor or prasugrel (relative risk [RR] 0.49, 95% CI 0.33–0.71) compared to clopidogrel and ASA.^[12]^ However, this meta-analysis is restricted by limited inclusion criteria, direct comparisons, and does not include more recent studies. For instance, most recently, Zhao et al evaluated P2Y12 inhibitors in all CABG patients. After surgery, they randomized CABG patients to ASA, ticagrelor, or DAPT with ticagrelor and ASA. SVG patency was improved with DAPT compared to ASA only (80.6% vs 89.9%, *P* = .006) with no significant difference in major bleeding.^[13]^

Despite encouraging data, evidence examining the optimal antiplatelet therapy choice after CABG is lacking. RCTs comparing DAPT with antiplatelet monotherapy in the CABG population are limited by small sample sizes or focus on patients undergoing CABG after an ACS only.^[12,13]^ Given the variety of antiplatelet agents available, along with the different possible combinations, we propose to conduct a multiple treatment comparison meta-analysis (a network meta-analysis [NMA]) evaluating the efficacy and harms of DAPT in patients following CABG. Different antiplatelet agents will be compared using direct and indirect data.^[14]^ Our question is: What are the comparative effects (in terms of SVG patency, mortality, MACE, and major bleeding) of different antiplatelet agents on patients after CABG.

## 2 Methods

We will conduct the systematic review and NMA in adherence with the Preferred Reporting Items for Systematic Reviews and Meta-Analyses (PRISMA) Extension for NMA of healthcare interventions guidelines.^[15]^

We registered our review and NMA protocol with the International Prospective Register for Systematic Reviews (PROSPERO)—CRD42019127695 **Eligibility criteria** (studies have to meet all inclusion criteria and none of the exclusion criteria to be included).

### 2.1 Types of participants

Adult patients (18 years or older) following any CABG surgery (including on and off-pump cases, minimally invasive and conventional sternotomy cases, any types and number of venous, and/or arterial grafts).

### 2.2 Types of studies

Parallel-groups RCTs (including factorial design RCTs).

### 2.3 Types of interventions

Any antiplatelet therapy or combination of antiplatelet therapies administered within 1 month after CABG and continued for a minimum of 3 months. Given the variation in ASA dosing, we will divide ASA into high-dose (>325 mg daily) or low-dose (≤325 mg daily). The intervention groups will include but may not be limited to: ASA, clopidogrel, prasugrel, ticagrelor, cangrelor, clopidogrel + ASA, prasugrel + ASA, ticagrelor + ASA, cangrelor + ASA, with placebo or no antiplatelet agent as a node as well.

### 2.4 Types of outcome measures

#### 2.4.1 All-cause mortality

We will evaluate mortality in-hospital, within 30 days, within 6 months, at 1-year, and at latest follow-up.

#### 2.4.2 SVG patency

Assessed by either CT angiography or catheter-based angiography at any time during the observation period of the study. If data are available, we will evaluate graft patency rates at any time within 3 months, within 6 months, at 1 year, and at latest follow-up.

#### 2.4.3 MACE

This will be a composite of all-cause mortality, stroke, systemic thromboembolic events, and myocardial infarction (MI). Whenever data are available, we will evaluate MACE at any time within 3 months, within 6 months, at 1 year, and at latest follow-up.

#### 2.4.4 Major bleeding

This will be defined as per individual study criteria. Whenever data are available, we will evaluate major bleeding rates at any time within 3 months, within 6 months, at 1 year, and at latest follow-up.

### 2.5 Search methods for identification of studies

#### 2.5.1 Electronic searches

We will search Cochrane Central Register of Controlled Trials (CENTRAL), Medical Literature Analysis and Retrieval System Online (MEDLINE), Excerpta Medica Database (EMBASE), Cumulative Index to Nursing and Allied Health Literature (CINAHL), American College of Physicians Journal Club (ACPJC) from inception to January 2019. Our search strategy was created in discussion with a librarian to ensure use of appropriate and broad Medical Subject Headings (MeSH) terms, to be as inclusive in the title and abstract phase as possible (Appendix 1).

#### 2.5.2 Searching other resources

We will review the reference lists of all included full-text articles and previous meta-analyses. We will review clinical trials data registry (clinicaltrials.gov, ISRCTN Register, and WHO ICTRP) for registered published or unpublished studies. We will search conference proceedings for the European Society of Cardiology Congress, American Heart Association Scientific Sessions, American College of Cardiology Conference, and Canadian Cardiovascular Congress within the last 2 years.

### 2.6 Data collection and analysis

#### 2.6.1 Selection of studies

We will perform title and abstract screening independently and in duplicate using the Covidence online software.^[16]^ If either reviewer deems a study relevant, it will be retrieved for full-text review. Full-text review will also be done in duplicate. We will resolve disagreements through discussion or third-party arbitration.

#### 2.6.2 Data collection and management

We will carry out data extraction independently and in duplicate using pre-piloted forms. If there is discrepancy, a third-party reviewer will assess the data.

#### 2.6.3 Risk of bias assessment for randomized controlled trials

Risk of bias will be evaluated as either low risk or high risk using the Cochrane Collaboration tool.^[17]^ Two independent reviewers will analyze each trial in six domains: random sequence generation, allocation concealment, blinding of participants and personnel, blinding of outcome assessment, selective reporting, and other sources of bias. If all aspects are considered to have low or probably low risk of bias, the study will be considered at low risk. If even one aspect or more has probably high risk of bias, the study will be considered at high risk.

### 2.7 Data extraction

Bibliometric information: Author, year of publication, countries where studies were conducted, publication journal, contact information, and funding source (s).

#### 2.7.1 Details of population

Mean age, sex, past medical history (hypertension, diabetes, dyslipidemia, previous MI, congestive heart failure, peripheral vascular disease, ACS presentation), surgical details (off-pump CABG, on-pump CABG, concomitant procedures).

Methodology: Design of RCT; risk of bias

Details of intervention and comparator: doses and frequencies, different comparisons made.

Details of outcome: mortality, SVG patency, major bleeding and criteria used to assess major bleeding, MACE (including mortality, MI, stroke, and systemic thromboembolic events).

### 2.8 Statistical analysis: indirect and mixed comparisons

We will perform conventional meta-analyses with a random effects model for all direct comparisons, and then a random effects Bayesian NMA in order to include both direct and indirect evidence. Since ASA monotherapy is the standard of care after CABG, we will use this as the reference for baseline risk, with the data originating from the ASA monotherapy arm of the RCTs.^[18,19]^ To assess the transitivity assumption, we will qualitatively assess patient distribution and evaluate study characteristics that modify treatment effects across comparisons by presenting tabulated results of these characteristics. We will evaluate the consistency assumption statistically by performing a global test for inconsistency (design-by-treatment interaction model) and a local test (inconsistency plot). We will report results as odds ratios with corresponding 95% credible intervals (95% CrI)—a Bayesian analog of 95% confidence intervals (CIs).^[15]^ We will run all models for a minimum of 100,000 iterations to ensure convergence. We will analyze data using R version 3.5.3.^[20]^ We will perform meta-regression to see if the length of follow-up is correlated with the outcome.

#### 2.8.1 Direct comparisons

We will use Review Manager 5.3 to perform the analyses.^[21]^ We will use a random effects model (DerSimonian and Laird method) as we expect heterogeneity among the studies due to differences in methodology.^[22]^ We will present the pooled results as RR with 95% CIs for dichotomous outcomes and as mean difference (MD) with 95% CI for continuous outcomes. Before pooling outcomes, we will assess the clinical and methodological heterogeneity, including population characteristics, definition, and utilized assessment tools. Should they be inappropriate for pooling, we will describe the results of each study independently.

#### 2.8.2 Multiple treatment groups

Studies with multiple treatment arms will be included and treated as multiple two-arm studies in a direct comparison. For the NMA, the patients in each treatment arm will be allocated to the respective node in the network.

#### 2.8.3 Treatment ranking

We will use the surface under the cumulative ranking curve (SUCRA) to estimate the probability of each intervention being ranked first. The more efficacious a treatment node, the higher the expected SUCRA score.^[23]^ We will also provide a rank of the treatment with the lowest overall (combined) complications.

#### 2.8.4 Dealing with missing data

Whenever appropriate, we will contact authors of primary studies to identify missed or unpublished data. We will assess and record how each trial handled missing data.

#### 2.8.5 Heterogeneity assessment

We will qualitatively assess heterogeneity by comparing the study population characteristics, interventions and outcomes of included trials within each pairwise comparison. To assess methodological heterogeneity, we will qualitatively compare the risk of bias within each pairwise comparison. We will assess statistical heterogeneity within each pairwise comparison using the *I*^2^ index, the Cochrane *Q*-test and visual inspection of the forest plots. We will assume a common heterogeneity variance (τ^2^ estimated using restricted maximum likelihood approach, across the different comparisons in the network).

#### 2.8.6 Publication bias

For direct comparisons in each outcome, we will inspect the funnel plots for publication bias by evaluating asymmetry if 10 or more studies are pooled.^[24]^ We will use an Egger's regression test for assessment if we identify possible publication bias.^[25]^ In order to assess for small-study effect within the network, we will use a comparison-adjusted funnel plot.^[26]^

#### 2.8.7 Assessment of certainty in pooled effect estimates

We will use the Grading of Recommendations Assessment, Development and Evaluation (GRADE) approach to evaluate the certainty of the evidence for each outcome.^[27]^ Our confidence assessment will address the risk of bias in individual studies, imprecision, inconsistency (heterogeneity in estimates of effect across studies), indirectness (related to the question or due to intransitivity), and publication bias.^[28]^ The certainty in indirect estimates will be inferred by examining the dominant first-order loop associated with the particular comparison and will be the lowest of the direct estimates contributing to the indirect comparison. If there are issues with intransitivity (important difference between studies forming the indirect loop), we will further lower the certainty in the indirect estimate. If there is incoherence between direct and indirect estimates, we will include the one with the higher certainty rather than the network estimate. For certainty in NMA estimates, we will use the higher of the direct and indirect (assuming they are coherent). Imprecision will be assessed at the NMA level and not at the level of the direct or indirect estimate.^[29]^

#### 2.8.8 Subgroup analysis and investigation of heterogeneity

We plan to have two subgroups: post-ACS versus not post-ACS and off-pump CABG versus on-pump. We hypothesize that post-ACS population will show a greater benefit with the different DAPT regimens than antiplatelet monotherapy. Similarly, the off-pump CABG population will also show a greater benefit with the different DAPT regimens than the various monotherapies. If a sufficient number of studies report these outcomes to create a non-sparse NMA, we will perform the analysis for that subgroup.

## 3 Discussion

While several studies have demonstrated the benefit on graft patency of a more intense antiplatelet therapy regimen, especially with DAPT using ASA and ticagrelor, the available evidence for mortality and morbidity is restricted by the small sample size of RCTs and sub-studies of larger RCTs.^[12,13]^ This study aims to qualitatively and quantitatively synthesize the available evidence comparing the efficacy and safety of different antiplatelet agents among patients undergoing CABG. Although this will not be the first review to evaluate the relative effects of multiple antiplatelet agents among CABG patients using an NMA approach, it will be the first to evaluate graft patency, mortality, MACE and major bleeding as protocolized outcomes of interest.^[30]^

Our review will have several strengths. First, we will conduct a comprehensive literature search to identify published and unpublished studies, restrict the evidence to RCTs, perform duplicate assessment of eligibility, risk of bias, and data extraction. Furthermore, we will use the GRADE framework to assess the quality of evidence. Our study will also have limitations; due to a broad range of publication dates (potentially from 1980s onwards), some of the studies may not reflect current clinical and surgical practices or expected outcomes. We also expect there to be significant variation in definition of MACE and major bleeding between studies. The analysis will also be limited in terms of subgroup analyses by using study-level data.

Despite these limitations, our NMA will be the largest quantitative synthesis assessing antiplatelet therapy among patients after CABG surgery to date. It should inform clinicians and guideline developers in selecting the most effective and safest antiplatelet regimen. Our study may also highlight gaps in the evidence on this topic, triggering further research to improve patient-important outcomes.

## Acknowledgments

None.

## Author contributions

SG made substantial contributions to conception and design of the work, drafted the work, and substantially reviewed it. EBC and RW made substantial contributions to conception of the work, and substantially reviewed it. BR made contributions to the design and methodology of the work, and substantially reviewed it. AB, PP, and AP made contributions to the methodology of the work. LM, SM, and JS made revisions to the final work. All authors read and approved the final manuscript.

**Conceptualization:** Saurabh Gupta, Emilie P Belley-Côté, Bram Rochwerg, Richard P Whitlock.

**Data curation:** Saurabh Gupta, Emilie P Belley-Côté, Puru Panchal, Arjun Pandey.

**Formal analysis:** Saurabh Gupta, Anthony Bozzo.

**Funding acquisition:** Saurabh Gupta, Emilie P Belley-Côté, Richard P Whitlock.

**Investigation:** Saurabh Gupta, Emilie P Belley-Côté, Bram Rochwerg.

**Methodology:** Saurabh Gupta, Emilie P Belley-Côté, Bram Rochwerg, Anthony Bozzo, Lawrence Mbuagbaw, Richard P Whitlock.

**Project administration:** Saurabh Gupta, Emilie P Belley-Côté, Richard P Whitlock.

**Resources:** Saurabh Gupta, Emilie P Belley-Côté, Bram Rochwerg, Richard P Whitlock.

**Software:** Saurabh Gupta, Anthony Bozzo.

**Supervision:** Emilie P Belley-Côté, J-D Schwalm, Richard P Whitlock.

**Validation:** Saurabh Gupta, Emilie P Belley-Côté, Richard P Whitlock.

**Visualization:** Saurabh Gupta, Emilie P Belley-Côté, Richard P Whitlock.

**Writing – original draft:** Saurabh Gupta.

**Writing – review & editing:** Saurabh Gupta, Emilie P Belley-Côté, Bram Rochwerg, Anthony Bozzo, Puru Panchal, Arjun Pandey, Shamir Mehta, J-D Schwalm, Richard P Whitlock.

Saurabh Gupta orcid: 0000-0002-0114-0027.

## Supplementary Material

Supplemental Digital Content
