# Supplementary material for: Antiplatelet therapy and coronary artery bypass grafting: Protocol for a systematic review and network meta-analysis
Source: Medicine (Baltimore). 2019 Aug 23;98(34):e16880. doi: 10.1097/MD.0000000000016880 (PMC6716708; doi:10.1097/MD.0000000000016880)
Supplement: Supplemental Digital Content [file medi-98-e16880-s001.docx]

| **APPENDIX 1.** MeSH terms used for search strategy.  1. exp Platelet Aggregation Inhibitors/ |  |
| --- | --- |
| 2. (anti-platelet* or antiplatelet).mp. |  |
| 3. ((platelet* or thromboxane or adenosine diphosphate receptor* or ADP receptor* or thienopyridene or cyclo-oxygenase or cyclooxygenase or cyclic GMP phosphodiesterase type V enzyme*) adj2 (inhibitor* or antagonist* or anti-aggrega*)).mp. |  |
| 4. exp Fibrinolytic Agents/ |  |
| 5. (fibrinolytic or antithrom* or anti-thromb* or thrombolytic).mp. |  |
| 6. (aspirin* or ASA or ticlopidine).mp. |  |
| 7. (acetylsal* or acylpyrin or aloxiprimum or aspirin or colfarit or dispril or easprin or ecotrin or endosprin or magnecyl or micristin or polopir* or solprin or solupsan or zorprin).mp. |  |
| 8. exp DIPYRIDAMOLE/ |  |
| 9. (dipyridamole or curantil or curantyl or dipyramidole or kurantil or miosen or novo-dipiradol or persantin or persantine).mp. |  |
| 10. (clopidogrel or plavix or clopilet or grepid or iscover or zopya or zylagren or zylit).mp. |  |
| 11. Prasugrel Hydrochloride/ |  |
| 12. (prasugrel or effient or efient).mp. |  |
| 13. (ticagrelor or brilinta or brilique or possia).mp. |  |
| 14. (indobufen or ibustrin).mp. |  |
| 15. (ticlopidine or ticlid or ticlodix or ticlodone).mp. |  |
| 16. or/1-15 |  |
| 17. exp Coronary Artery Bypass/ |  |
| 18. ((coronary or aortocoronary or aorto-coronary) adj2 bypass*).mp. |  |
| 19. CABG*.mp. |  |
| 20. 17 or 18 or 19 |  |
| 21. 16 and 20 |  |
| 22. random*.mp. |  |
| 23. exp randomized controlled trial/ |  |
| 24. exp Randomized Controlled Trials as Topic/ |  |
| 25. Random Allocation/ |  |
| 26. double-blind method/ or single-blind method/ |  |
| 27. ((singl* or doubl* or tripl* or trebl*) adj3 (blind* or mask* or conceal* or procedure*)).mp. |  |
| 28. meta analys?s.mp. |  |
| 29. exp Meta-Analysis/ |  |
| 30. exp Meta-Analysis as Topic/ |  |
| 31. systematic review*.mp. |  |
| 32. or/22-31 |  |
| 33. 21 and 32 |  |
